# The effect of a prosocial environment on health and well-being during the first COVID-19 lockdown and a year later

**DOI:** 10.1038/s41598-024-56979-2

**Published:** 2024-03-19

**Authors:** Estherina Trachtenberg, Keren Ruzal, Oren Forkosh, Inbal Ben-Ami Bartal

**Affiliations:** 1 Sagol School of Neuroscience, Tel Aviv University, Tel Aviv, Israel; 2 School of Psychological Sciences, Faculty of Social Sciences, Tel Aviv University, Tel Aviv, Israel; 3 Department of Cognitive and Brain Sciences, Hebrew University of Jerusalem, Jerusalem, Israel; 4 Department of Animal Sciences, Hebrew University of Jerusalem, Jerusalem, Israel

**Keywords:** Psychology, Health care, Risk factors

## Abstract

The outset of the COVID-19 pandemic was characterized by prolonged periods of chronic stress and social isolation. While studies have investigated the changes to well-being (WB) during this period, the impact of the social environment on long-term physical and mental health requires further study. This study aimed to assess the factors influencing WB and health outcomes, with the hypothesis that a positive social environment would play a significant immediate and long-term role in improving WB and preventing the effects of anxiety associated with the pandemic. At time point 1 (April 2020), an Israeli sample of 206 participants (84% female, mean age 31.5) responded to traditional questionnaires assessing mental health and social support. Factors affecting WB were assessed within subjects during the first COVID-19 lockdown for 6 weeks using a daily survey (Beiwe phone application). A year later, in May 2021, at time point 2, the initial questionnaires were readministered to a subset of the same participants (N = 94). We found that anxiety during the first lockdown adversely affected WB and predicted health and WB deterioration a year later. In contrast, a high quality of social relationships was associated with better short- and long-term WB, and mitigated the adverse effects of anxiety. Daily activities, including physical activity, meditation, and romantic relations, were also positively associated with WB during the first lockdown but did not have long-term effects. In summary, our study underscores the enduring health advantages of a positive social environment, particularly during stressful periods. These results have implications for health policymakers: programs which support individuals with high anxiety and low support, by integrating them into community-based interventions, promise to enhance well-being (WB) and health, as well as to fortify the community as a whole.

## Introduction

The onset of the COVID-19 pandemic was marked by a period of chronic stress for vast populations across the world, with increased reported anxiety, loneliness, and perceived threat of contracting the virus^1–4^. Increased levels of anxiety, stress, and the risk of depression have also been recorded in Israel at the acute onset of the pandemic^5–8^. An increase in a sense of loneliness was also found, especially among young adults^9^. In a large-scale, 3-year longitudinal study conducted in Israel to assess anxiety in the population, researchers found significantly higher levels of anxiety and depression during the pandemic outbreak (mid-2020) compared to the first wave conducted before the pandemic (2017)^3^. However, while there have been several Israeli studies examining anxiety and mental health at the beginning of the pandemic, assessing the long-term effects of anxiety on mental and physical health remains an understudied area. Anxiety is considered a risk factor for various diseases, such as cardiovascular disease^10,11^. Additionally, during the pandemic, anxiety and loneliness have been found to be negatively related to general physical and mental health^12–14^. As the pandemic combined acute stress with a major disruption of the social environment, investigating the link between social support, well-being (WB) and health is of utmost importance.

In the first few months of the pandemic, which were characterized by enforced social distancing and strict quarantine, a rise in reports of decreased happiness levels and WB was observed^15–18^. The concept of WB represents diverse traits contributing positively towards one's happiness, satisfaction, and meaning in life^19,20^. WB has been strongly associated with positive health outcomes and quality of life^20^. It has been shown that the pandemic has significantly impacted healthcare worldwide^21^, which has resulted in general health deterioration, high financial costs, and high human costs^22^. Data collected pre-pandemic indicate that chronic stress and anxiety are linked to poorer health outcomes, with symptoms including sleep disturbances, fatigue, changes in appetite, increased inflammation, higher rates of viral infections, autoimmune disorders, and various other mental health issues^23–25^. Therefore, it is important to monitor changes to WB levels and health, especially in times of stress. As the negative impact on mental health during a pandemic carries significant societal and human costs, it is crucial to identify factors that promote resilience and WB. Emerging research points related to such factors include psychological traits like gratitude and extraversion^26–28^, and social factors including support and belongingness^29–32^. Social support refers to the help and emotional comfort provided by individuals, groups, or communities^33^. It serves as a protective factor for mental and physical health^34^.

A cross-sectional study evaluating social support during the pandemic reported that, even via online platforms, support was associated with greater WB^30^. A large-scale longitudinal study showed a significant negative correlation between the quality of social relationships (QSR) and depressive symptoms during the pandemic period^35^. QSR is a term used to encompass the positive aspects of relationships and emotional support provided by significant others. As social interactions can be negative, it is important to index their valence, not merely their occurrence. Factors such as satisfaction, acceptance, trust, commitment, and belongingness also heavily influence relationship quality^36^.

Not only receiving social support but also providing support has shown to be beneficial for WB during COVID-19. A large international cross-sectional study conducted in 78 countries during the pandemic demonstrated that self-reported engagement in prosocial behavior was associated with better WB^37^. Similarly, a cross-sectional study conducted using daily surveys reported that engaging in prosocial activities was associated with higher levels of positive affect and greater social satisfaction on the same day^38^.

Daily behavioral patterns and their impact on WB are often studied using cross-sectional and longitudinal methods. Cross-sectional and longitudinal studies face challenges in capturing real-time, contextual insights and dynamic changes, often leading to biases and static conclusions. An ecological momentary assessment approach, such as the experience sampling method (ESM), is advantageous for understanding daily fluctuating behaviors and subjective repeated factors. ESM allows for real-time data collection providing dynamic insight into human experiences in natural settings. Hence, the ESM is ideal for self-report on daily experiences over a prolonged period^39,40^.

ESM has been utilized in numerous studies across various populations to evaluate daily behavioral changes during the COVID-19 pandemic^41–43^. However, only a select few have focused on assessing WB during this period^44–46^. For instance, Stieger et al.'s assessment of emotional WB found that being outdoors was positively associated with WB, whereas reported loneliness had the opposite effect^46^. Although there is a body of research concentrating on social support throughout the pandemic, to our knowledge, only a limited number of studies have applied ESM to the daily assessment of WB throughout this period.

The long-term effects of the pandemic are beginning to emerge, yet the link between daily activities during the pandemic (e.g., social interactions, mood, sleep, entertainment, physical activity, etc.) and health outcomes has been relatively unexplored. This study addresses these gaps by measuring how QSR and prosocial behavior impacted the WB of Israelis during the first COVID-19 lockdown as well as a year later. In this study, we employed a mixed-methods approach, utilizing longitudinal methodology and ESM to understand how the social environment and prosocial behavior influence both WB and health during a period of heightened anxiety.

This study aimed to understand how the social environment, prosocial behaviors, anxiety, and daily events impacted (a) WB during the first COVID-19 lockdown in Israel and (b) the long-lasting effect on WB and health in the same participants a year later. It was hypothesized that WB levels would be negatively impacted by the lockdown at the study's onset^35,46^. Furthermore, improvement in WB was anticipated when lockdown restrictions were lifted. It was also posited that the negative effects of anxiety experienced during the lockdown on mental and physical health would be mitigated by a supportive social environment, both in the immediate and long term^47,48^.

**Figure 1:**
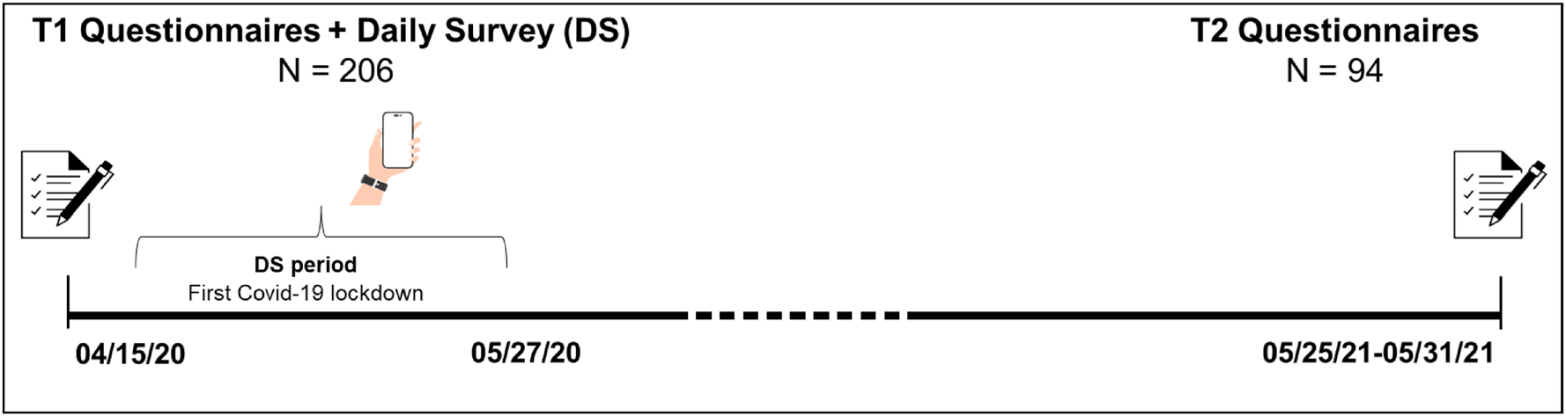
Outline of study design.

To address these questions, participants responded to questionnaires containing validated scales of mental health and social support at two time points (Fig. 1). The first time point ("T1") was assessed soon after the initiation of the first COVID-19 lockdown in Israel (April 2020). Additionally, daily reports were collected by means of a daily survey (DS) conducted over 43 days via the Beiwe application^49^. These participants were reassessed a year later ("T2", May 2021). By this time, the number of infections had decreased to nearly zero in Israel following a widespread public vaccination effort^50^, and restrictions had been completely lifted, including the mask mandate^51^. While a relapse would occur later that summer, during this time point, the public sentiment in Israel reflected a belief that the pandemic had ended^51^. This strategy provided a unique perspective into the state of mind of participants during the initial pandemic period in Israel, as well as the effect of these experiences on long-term health. Understanding these factors is needed for the development of effective interventions for use during future global threats.

## Materials and methods

### Participants

Participants were recruited via advertisements on social media such as Facebook and WhatsApp groups, which included a request to share the link with acquaintances. The advertisement contained a link to an online Google form, in which the participants signed a detailed consent form as approved by the ethics committee and all methods were performed in accordance with the relevant guidelines and regulations. Participants under the age 18 were excluded. The ethics committee of Tel Aviv University approved the study protocol, consent form, and advertisement (1257-1). Informed consent was obtained from all the participants. The consent form included the topic of the study, which stated the aim of improving WB during stressful times. Furthermore, their right to withdraw from the study at any time was emphasized. Participants were compensated via participation in a lottery for a small prize (book or restaurant coupon). Identities were encrypted and were unavailable to the experimenters analyzing the study.

### Study design

During the first weeks of the COVID-19 lockdown in Israel (April 15–May 27, 2020), participants responded to a participation ad published on social media. Participants gradually opted to join the study between April 13–16, after which no further participants were accepted. At T1, participants were asked for general demographics and responded to a set of validated questionnaires, including the Mental Health Continuum (MHC), the Multidimensional Scale of Perceived Social Support (MSPSS), and the Depression Anxiety Stress Scales (DASS). Altogether, T1 questions required approximately 10–15 min to complete, as was confirmed by investigators during pilot testing. After completing the T1 questionnaires online, participants were instructed on the installation of the Beiwe phone application. Subsequently, a daily survey (DS) was conducted over a 43-day period using Beiwe. The DS was concluded on May 28, when all restrictions were lifted by the Israeli government. Notifications were sent out daily to all participants at 7 PM. All responses provided before 12 AM were collected. The DS consisted of 13 questions assessing social interactions, mood, daily behaviors, and events. The mean DS response time was 3 min, with a range of 2–5 min. At T2 (May 25–31, 2021), 94 of the same participants responded to the same questionnaires as T1, along with additional health-related items—a health deterioration score and the 2-Way Social Support Scale (SSS). Initially, 608 participants responded to the T1 questionnaires; however, only 206 participants who downloaded the app and participated in the DS were included in this study (see Fig. 1 for the study timeline).

### Measurements

*Mental Health Continuum Short Form (MHC)*^52^*:* a 14-item questionnaire measuring three scales of emotional, psychological, and social scales of mental health assessment. The emotional scale measures happiness and life satisfaction, the psychological scale measures self-acceptance and personal growth, and the social scale measures social coherence and acceptance. The instructions were to answer the questionnaire based on the previous month's experience on a Likert scale of 6 points, where 1 indicates "never", and 6 indicates "every day". Higher MHC total scores indicated higher WB levels. The questionnaire has been translated and validated for the Hebrew language^53^. In the present study, Cronbach's alpha = 0.906.

*Depression, Anxiety, and Stress Scales (DASS)*^54^*:* a 21-item questionnaire that assesses symptoms of depression, anxiety, and stress. The depression scale describes low levels of positive affect, the anxiety scale represents physiological hyperarousal, and the stress scale contains characteristics such as irritability and agitation. The instructions were for participants to answer based on their experience the week before, on a Likert scale from 0 = did not apply to me at all, to 3 = applied to me very much or most of the time. A validated Hebrew version of the DASS questionnaire was used in this study^55,56^. In the present study, Cronbach's alpha = 0.88.

*Multidimensional Scale of Perceived Social Support (MSPSS)*^57^: a 12-item questionnaire that rates social support levels from family, friends, and significant others. The instructions for the questionnaire were for participants to respond based on what they "feel right now" on a Likert scale from 0 = very strongly disagree to 7 = very strongly agree. The questionnaire has been translated and validated for the Hebrew language^58^. In the present study, Cronbach's alpha = 0.91.

*2-Way Social Support Scale (SSS)*^59^*:* a 21-item questionnaire that measures receiving or giving emotional or instrumental social support. The instructions for the questionnaire were: "Please grade the following statements on a Likert scale ranging from 1 = never to 6 = always". Each dimension (giving-instrumental, giving-social, receiving-instrumental, receiving-social) has its own mean score. The "forwards and backwards" translation protocol^60^ was used to translate the SSS questionnaire to Hebrew by an experimenter and cross-validated by a second, independent experimenter translating it back to English. In the present study, Cronbach's alpha = 0.89.

*Health deterioration score:* we assembled a ten-item list to encompass the main symptoms known to be influenced by prolonged periods of stress. Our goal was to measure potential health deterioration resulting from the past year, marked by multiple lockdowns and elevated stress levels. Participants were asked as follows: "Compared to the year prior to the COVID-19 outbreak, have you experienced during the recent year any of the following health problems, and if so, did these problems reflect a worsening of an existing condition, or rather a new condition?". The response items included difficulties sleeping or falling asleep, fatigue or low energy, low appetite or overeating, weight loss or weight gain, difficulties concentrating, suicidal thoughts or fears about the future, abnormal blood-test results, mental health issues (e.g., anxiety or depression), chronic diseases (e.g., autoimmune or skin conditions), frequent colds and flu, and reduced sexual drive. Participants were then asked to rate these experiences using a Likert scale as follows: 1 = improved greatly, 2 = improved slightly, 3 = no change, 4 = deteriorated slightly, 5 = deteriorated greatly, 6 = a new condition. Participants could select multiple items or choose not to select any items if they had not experienced any of these conditions.

*Composite scores:* in order to assess the changes from T1 to T2, a composite score representing WB for T1 and T2 was generated by combining the answers from DASS (anxiety, stress, depression), MHC (mental health), and MSPSS (social support) for each of these periods. This composite score was a more comprehensive and well-rounded measure of WB, as it considered positive and negative aspects of WB and social support. To arrive at the composite score, the means were standardized to a scale between 0 and 1, and the composite was then computed as MHC + MSPSS-DASS, such that higher scores represented higher reported WB. The composites for T1 WB and T2 WB received Cronbach's alpha scores of 0.74 and 0.71, respectively. The delta between T2 and T1 scores (Delta_WB) was calculated as T2 WB-T1 WB.

A personality questionnaire (BFI) was also administered during T1. However, as this study focused on the effect of environmental factors (social and non-social) on the response to COVID-related stress, the investigation of personality traits was not pursued for this study and is not discussed.

Daily survey (DS) items and procedures: the DS assessment included 13 items across four main topics: WB, anxiety, the social environment, and daily events and habits. Given the expectation that participants complete the DS daily over several weeks, we aimed to make it brief and precise to minimize the risk of participant attrition. Consequently, we developed a set of items drawing from the validated questionnaires used in this study (MSPSS, DASS, and MHC). The DS includes three WB items (mood, happiness, vigor), one anxiety item, and four questions regarding the quality and quantity of social interactions. Furthermore, five items assess daily events (e.g., sleep, sports, entertainment). See Table 1 for detailed DS items. The four main DS factors (DS WB, anxiety, QSR, and loneliness) showed significant correlations (ranging from moderate to high, p < 0.0001) with scores from the validated questionnaires, reinforcing the DS's validity. See Table S1 in the supplementary materials for the correlation data.

**Table 1:** Daily survey (DS) items .

Question number	Daily survey questions	Answer scale	Composite score
1	Mood: How was your mood today?	0 = *very bad*; to 4 = *excellent*	Daily WB (DS WB) = Mood + Happiness + Vigor, max out of 8 Cronbach's alpha = 0.77
2	Happiness: Did you laugh or smile today?	0 = *not at all*; to 2 = *a lot*	
3	Vigor: How much vigor or motivation did you have today?	0 = *not at all*; to 2 = *a lo*t	
4	Quality of social relationships (QSR): How did you experience the relationships with people around you today?	1 = *horrible*; to 5 = *very pleasant*	
5	Anxiety: What are your anxiety levels today?	0 = *not experiencing anxiety*; to 4 = *very high levels*	
6	Loneliness: Are you feeling lonely today?	0 = *no*; 1 = *yes*	
7	People with you: How many people were physically with you?	0 = no one; to 4 = more than 10 people	
8	Social interactions: How many interactions over the phone/social media/face to face did you have today?	0 = with no one; to 4 = with more than 10 people	
9	Prosocial behavior (giving): Did you do anything nice for other people?	0 = *no*; 1 = *yes*	
10	Prosocial behavior (receiving): Did anyone do anything nice for you?	0 = *no*; 1 = *yes*	
11	Sleep: How many hours did you sleep last night?	0 = 0–2 h; to 6 = more than 12 h	
12	Daily habits (sports): Please check the box if you did one of the following activities today	Yoga, walk the dog, run/walk outside, aerobic activity, other	Sports = sum of the activities
13	Daily habits (substances)—please check the box if you used one of the following substances today:	Alcohol, drugs, anti-depression/anxiety pills, chocolate	Substance = sum of the uses
	Other daily habits: Please check the box if you did one of the following activities today	Meditation, romantic relations, read a book, dance /listen to music, watch a movie	Individual score 0/1 for each component

Beiwe smartphone application: the Beiwe research platform^49^ was used to collect daily survey (DS) questions for 43 days. Beiwe is an open-source end-to-end encrypted digital platform for data storage and processing that includes an application for Android and iOS devices. Participants received a daily prompt inviting them to respond to the DS. Responses were encrypted on the phones and storage server. GPS information or other passive data options were not collected. Data collection complied with local data protection laws and regulations. Information on Beiwe is available here: https://beiwe.wpengine.com/about/.

### Statistical analyses

Complete statistical data are presented in the results section; means are reported as mean ± SEM and presented in Table 3. Statistical analyses described below were performed using R, SPSS, and GraphPad Prism.

T1 and DS analyses were performed on 206 participants. Participants with less than five consecutive days were excluded from DS repeated analysis. A year later, 94/206 participants responded to T2. A missing-data pattern analysis found that attrition was not related to any of the T1 variables. However, due to the large attrition rate (55%), we chose not to impute missing data. Therefore, all the analyses related to T2, association, and prediction from the DS parameters were performed on these 94 participants only. Chi-squared and t-test analysis was employed to test the difference in demographic characteristics between T1 and T2 (Table 2).

**Table 2:** Demographic characteristics of the sample.

	T1 + DS (N = 206)	T2 (N = 94)	Χ^2^ test
Mean age (STD)	31.5 (9.5)	33.1(10.5)	p = 0.20 (t-test)
Gender % (n)			
Male	16% (34)	14% (13)	p = 0.55
Female	84% (172)	86% (81)	
Family status % (n)			
Single	52% (106)	52% (49)	p = 0.92
Partnered	20% (41)	17% (16)	
Married	26% (54)	29% (27)	
Divorced	2% (5)	2% (2)	
Having kids % (n)			
Yes	77% (159)	76% (71)	p = 0.75
No	23% (47)	24% (23)	
People in the house % (n)			
Alone	11% (22)	12% (11)	p = 0.65
2	33% (68)	34% (32)	
3	21% (44)	24% (23)	
4	17% (35)	18% (17)	
5+	18% (39)	12% (11)	
Economic status % (n)			
Hardship	1% (2)	1% (1)	p = 0.94
Unstable	20% (41)	27% (16)	
Stable	51% (105)	53% (50)	
Comfortable	28% (58)	29% (27)

**Table 3:** Scores of all questionnaires and daily surveys.

Time point	Score/variable	Mean	Range	SD	SEM
T1 Questionnaire	MHC total	3.78	1.6 to 6	0.79	0.055
	MSPSS total	5.67	1 to 7	1.13	0.079
	DASS anxiety	3.05	0 to 17	3.51	0.245
	DASS depression	7.24	0 to 21	5.26	0.367
	DASS stress	8.57	0 to 20	4.70	0.328
	DASS total	19.06	0 to 55	11.12	0.775
	WB (T1 WB)	0.3402	− 0.11 to .61	0.144	0.010
Daily survey (DS) Beiwe app	WB (DS WB)	5.2	2.06 to 7.83	1.10	0.076
	Anxiety	0.750	0 to 2.9	0.694	0.048
	Loneliness	0.177	0 to 1.0	0.237	0.016
	QSR	3.504	1 to 5.0	0.754	0.053
	People around	2.06	0 to 3.8	0.684	0.048
	Prosocial behavior, giving	0.494	0 to 1	0.283	0.019
	Prosocial behavior, receiving	0.492	0 to 1	0.303	0.021
	Social interactions	2.21	1 to 4	0.759	0.052
	Sleep	3.14	1 to 4.6	0.539	0.038
	Sport	0.719	0 to 2.2	0.455	0.031
	Substance use	0.789	0 to 3	0.534	0.037
	Meditation	0.068	0 to 1	0.185	0.012
	Movie	0.363	0 to 1	0.301	0.021
	Music/dance	0.430	0 to 1	0.315	0.022
	Book	0.170	0 to 1	0.251	0.017
	Romantic relations	0.109	0 to 1	0.149	0.010
T2 Questionnaire	MHC total	3.78	1.9 to 6	0.901	0.092
	MSPSS total	5.67	1.8 to 7	1.18	0.122
	DASS anxiety	3.56	0 to 21	4.64	0.478
	DASS stress	7.74	0 to 21	5.15	0.532
	DASS depression	5.94	0 to 21	5.24	0.540
	DASS total	17.8	1 to 20	12.5	10.29
	WB (T2 WB)	0.347	− 0.1 to .6	0.155	0.016
	Delta WB (T2 WB—T1 WB)	0.001	− .27 to .36	0.127	0.013
	SSS-2—receiving and giving emotional and instrumental support				
	Receiving emotional	4.93	2 to 6	1.24	0.129
	Giving emotional	4.83	1 to 6	1.15	0.119
	Receiving instrumental	4.86	2 to 6	1.11	0.115
	Giving instrumental	4.10	1 to 6	0.98	0.101
	Mental health				
	Sleep	3.64	2 to 6	0.897	0.093
	Tired	3.87	2 to 6	0.958	0.099
	Eat	3.72	2 to 6	0.976	0.102
	Concentrate	3.66	2 to 6	0.905	0.094
	Sex drive	3.29	2 to 6	0.833	0.087
	Worries	3.70	2 to 6	0.899	0.094
	Anxiety/Dep	3.45	2 to 6	0.776	0.081
	Physical health				
	Blood	3.18	2 to 6	0.569	0.060
	Chronic disease	3.12	2 to 6	0.491	0.051
	Flu	3.07	2 to 6	0.467	0.049
	Health sum	3.29	2 to 10.00	2.56473	0.26595

DS repeated measure variables analyses: (R v4.2.0, R Development Core Team, 2021) WB was modeled in two separate hierarchical linear models (HLM), using the *lmer*() function ("Linear Mixed Effects in R") from the lme4 package^61^. Model A (Table 4) predicts DS WB by means of all the daily questions regarding emotional and social states, including DS anxiety, loneliness, quality of social interactions (QSR), number of social interaction, and related questions. Model B (Table 5) predicts DS WB by means of all the daily habit questions as yes/no answers (sports, dance, books, substance use, etc.). DS questions were categorized as one of two variables: (1) trait effect, a participant’s average score across the DS period and (2) state effect*,* which is the participant’s daily deviation from their average (i.e., their trait). The overall effects reflect the trait, while the daily effects reflect the within-person state, allowing for a more comprehensive understanding of both between and within-participant associations. We employed pseudo-standardized coefficients to enhance the comparability of different effects in our mixed model. Unlike unstandardized effect sizes, which can vary widely, pseudo-standardized coefficients provide a more uniform scale for comparison^62^. This method is commonly used in mixed model analysis and ESM analysis^63–65^. For the DS comparisons of D1 to the last day, multiple t-tests were conducted and corrected with FDR. Association between DS parameters and T2: to examine the predictors from DS and their effect on T2 WB, a simple linear regression was performed using the *lm*() function ("Linear Fit Model R"). Model C (Table 6) predicts T2 WB by means of all the overall (average) DS questions regarding emotional and social states (anxiety, loneliness, QSR, number of social interactions, etc.). The direct effects of DS anxiety, QSR, and loneliness on health deterioration a year later (T2) were examined via linear regression. To examine the indirect effect of how QSR and loneliness affect health deterioration a year later, we used a pathway (mediation) analysis using the *mediate*() function from the mediation package^66^.

**Table 4:** Model A. DS factors (anxiety and social environment) predicting DS WB.

Predictors	Std. Coef	SE	95% CI	t(169.70)	p
(Intercept)	0.00	0.00	0.00, 0.00	4.73	< 0.001
Trait effects (between/overall effect)					
Loneliness	− 0.16	0.06	− 0.29, − 0.03	− 2.50	**0.01**
Anxiety	− 0.23	0.06	− 0.34, − 0.12	− 4.06	**0.00**
Prosocial giving	0.06	0.08	− 0.09, 0.21	0.83	0.41
Prosocial receiving	0.02	0.08	− 0.14, 0.17	0.20	0.84
Social interactions	0.05	0.06	− 0.08, 0.17	0.72	0.47
People around you	0.08	0.06	− 0.04, 0.20	1.26	0.21
QSR	0.51	0.06	0.38, 0.63	7.89	**0.00**
Romantic relations	0.11	0.06	0.00, 0.22	2.05	**0.04**
State effect (within/daily effect)					
Loneliness	− 0.18	0.01	− 0.21, − 0.15	− 12.52	**0.00**
Anxiety	− 0.21	0.02	− 0.25, − 0.17	− 11.44	**0.00**
Prosocial giving	0.08	0.01	0.05, 0.10	5.95	**0.00**
Prosocial receiving	0.02	0.01	− 0.01, 0.04	1.11	0.27
Social interactions	0.08	0.02	0.05, 0.11	4.98	**0.00**
People around you	0.07	0.02	0.03, 0.11	3.83	**0.00**
QSR	0.45	0.02	0.40, 0.50	18.17	**0.00**
Romantic relations	0.07	0.01	0.05, 0.10	5.98	**0.00**

R^2^ Conditional = 0.703, R^2^ marginal = 0.478.

Linear mixed model fit by REML. t-tests use Satterthwaite's method, for each coefficient, we standardized by multiplying by SDx/SDy.

Significant values are in [bold].

**Table 5:** Model B. DS factors (behavioral patterns, and daily events) predicting DS WB.

Predictors	Std. Coef	SE	95% CI	t(169.70)	P
(Intercept)	0.00	0.00	0.00, 0.00	22.60	< .001
Trait effects (between/overall effect)					
Sport	0.17	0.08	0.00, 0.33	2.01	**0.05**
Substance	− 0.10	0.08	− 0.26, 0.05	− 1.29	0.20
Meditation	0.18	0.08	0.02, 0.34	2.18	**0.03**
Movie	0.10	0.08	− 0.06, 0.25	1.19	0.23
Music/dance	0.06	0.08	− 0.11,0.22	0.71	0.48
Book	− 0.03	0.08	− 0.19, 0.13	-0.39	0.70
Sleep	0.00	0.08	− 0.16, 0.15	-0.03	0.97
State effect (within/daily effect)					
Sport	0.11	0.02	0.07, 0.15	5.72	**0.00**
Substance	0.11	0.02	0.07, 0.15	5.47	**0.00**
Meditation	0.03	0.02	0.00, 0.07	1.95	**0.05**
Movie	0.00	0.02	− 0.04, 0.04	0.08	0.94
Music/dance	0.11	0.02	0.08, 0.15	6.76	**0.00**
Book	0.01	0.02	− 0.03, 0.05	0.32	0.75
Sleep	0.00	0.02	− 0.04, 0.04	-0.04	0.97

R^2^ Conditional = 0.481, R^2^ marginal = 0.064.

Linear mixed model fit by REML. t-tests use Satterthwaite's method, for each coefficient, we standardized by multiplying by SDx/SDy.

Significant values are in [bold].

**Table 6:** Model C. DS factors predicting T2 WB.

Predictors	Std. Coef	SE	95% CI	t(84)	p
(Intercept)	0.00	0.00	0.00, 0.00	1.99	**0.05**
DS average					
Anxiety	− 0.38	0.09	− 0.56, − 0.21	− 4.31	** < 0.001**
Loneliness	− 0.21	0.10	− 0.41, − 0.02	− 2.27	**0.033**
Social interactions	0.15	0.11	− 0.06, 0.37	1.42	0.160
People around	− 0.16	0.10	− 0.36, 0.04	− 1.63	0.106
Prosocial behavior—giving	− 0.16	0.13	− 0.42, 0.10	− 1.24	0.217
Prosocial behavior—receiving	0.23	0.13	− 0.02, 0.48	1.83	0.070
QSR	0.27	0.10	0.07, 0.47	2.66	**0.009**
Sleep	0.01	0.08	− 0.15, 0.18	0.16	0.870

R^2^ Conditional = 0.465, R^2^ adjusted = 0.422.

Simple linear regression, was standardized by multiplying each coefficient by SDx/SDy.

Significant values are in [bold].

SPSS v26.0 and GraphPad Prism v9.0 were used to perform the following tests: paired and unpaired t-tests, as well as one-way and two-way ANOVAs, were used to compare means between groups, including all demographic variables, high vs. low QSR levels, DS anxiety categories (mild, moderate, severe), reliance on friends (yes, no) and T1 WB categories (low, others), weekend effect (weekdays vs. weekends), and DS period (first vs. last day).

Standardized regression coefficients (Beta) with their 95% confidence intervals (CI) were reported. Variance inflation factors (VIF) were used to check for multicollinearity between predictors in all analyses and were found to be low (all VIF < 2.3). The level of significance was determined by p < 0.05. Full R scripts are presented in Supplementary materials.

For the composite scores (DS WB, T1 WB, T2 WB), Cronbach's alpha-internal consistency was performed as presented in “Materials and methods” section.

## Results

### Assessment of well-being during the first lockdown

To investigate how participants' WB was impacted during the first lockdown, a composite score was constructed based on the MHC, DASS, and MSPSS questionnaires (referred to as the "T1 WB score," as detailed in “Materials and methods” section). A total of 206 Israeli participants took part in the study (n = 172 females; n = 34 males), age (mean ± STD): 31.5 ± 9.5, ranging from 19 to 68 years. Of these, 51% were single, 20% were in a relationship, and 26% were married, for detailed demographics, see Table 2. In the DASS scales, a mean total of 19.06 ± 0.78 was reported, and the means for stress, anxiety and depression subscales placed the participants on mild to moderate levels according to the questionnaire's classification^46^. The means for T1 WB, as well as each sub-component, are fully detailed in Table 3.

**Figure 2:**
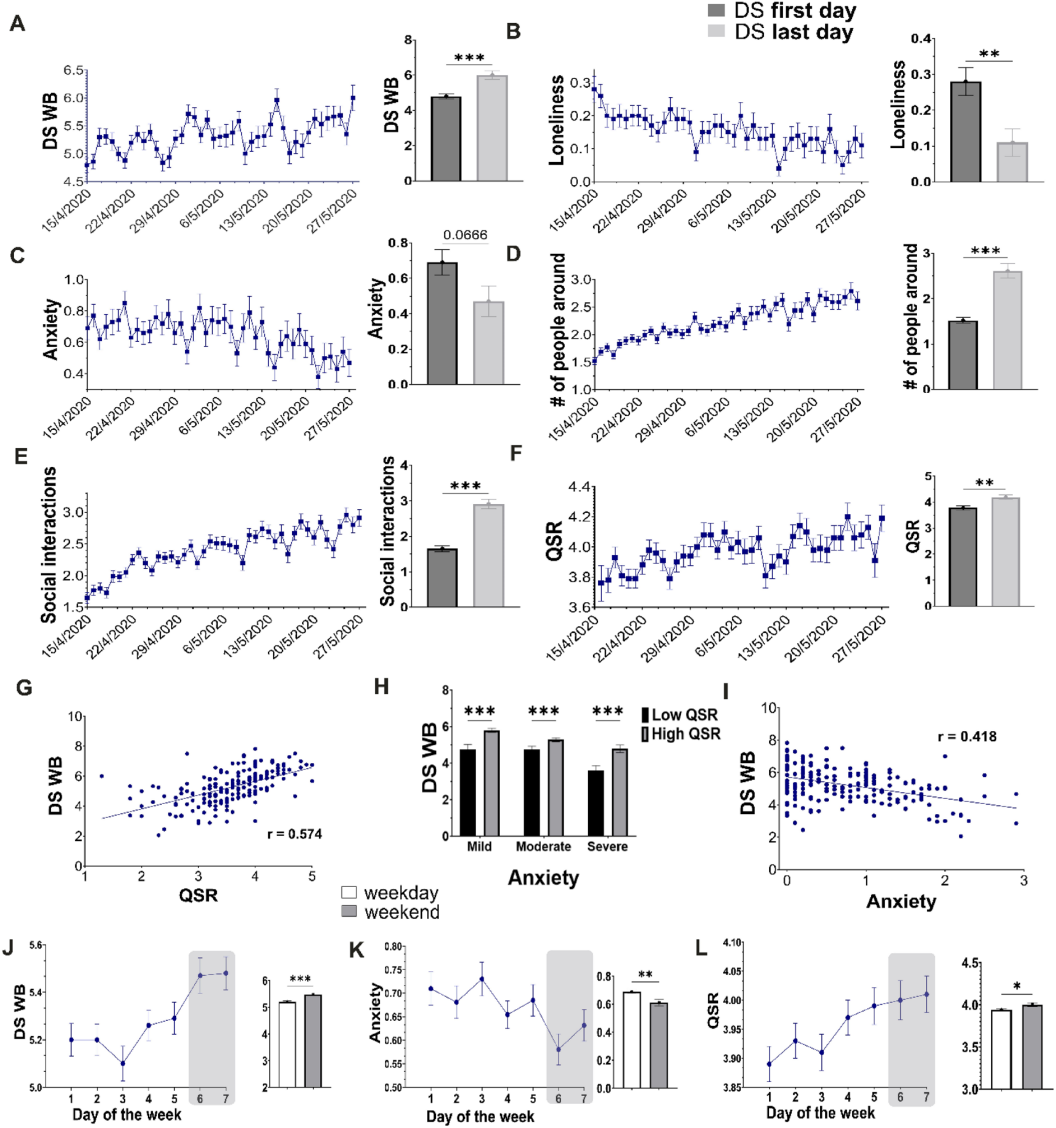
Factors associated with WB during the lockdown period. (**A**–**F**) Significant changes were observed to WB and the social environment along the lockdown period. Graphs show daily mean levels during the DS period (left), and a comparison between the first and the last day of the DS (right), showing improvement in these parameters along the DS period. (**G**) A significant positive association was observed between QSR and DS WB. (**H**) QSR significantly impacted DS WB across all DS anxiety levels. (**I**) DS anxiety was negatively associated with DS WB. (**J–L**) Improvement in DS WB, DS anxiety and QSR was observed during weekend days compared to weekdays (left). The bar graphs represent the mean levels of the first DS Day compared to the last DS Day. All error bars represent the standard error of the mean (SEM). *p < 0.05, **p < 0.001, ***p < 0.0001.

A demographics analysis revealed several parameters that influenced T1 WB levels. T1 WB was not significantly influenced by gender (ANOVA, F(1,204) = 0.7 p = 0.78) or age (ANOVA, F(3,202) = 1.8 p = 0.14). Marital status was associated with WB; single participants presented significantly lower T1 WB scores compared to participants with partners (ANOVA, F(3,202) = 5.9 p > 0.001, LSD post hoc p < 0.05), and participants with children had significantly higher T1 WB scores compared to childless participants (ANOVA, F(1,204) = 4.6 p = 0.03). Finally, participants with low economic status ("hardship" and "unstable") presented lower mean T1 WB scores compared to other economic groups ("stable" and "comfortable", ANOVA, F(3,203) = 10.4 p > 0.001, LSD, p < 0.05, see Table 2 for detailed demographics). In addition to answering the T1 questionnaires, participants responded to the DS regarding their affective state, social support, and daily activities (Fig. 1, Table 1) for up to 43 days. A daily WB score for this 6-week period was constructed based on the DS sum of reported mood, happiness, and vigor ("DS WB," Table 1, see ““Materials and methods””). Analysis of the change in DS WB between the first and last DS day revealed a significant increase in DS WB levels (Fig. 2A, t(195) = 4.77, p < 0.001). Furthermore, loneliness levels dropped significantly (Fig. 2B, t(196) = 2.75, p < 0.05), and DS anxiety levels marginally decreased (Fig. 2C, t(196) = 1.85, p = 0.06). This improvement may reflect easing social-distancing restrictions that coincided with the DS dates. Indeed, by the end of the DS period, participants reported a significantly higher number of people around them (Fig. 2D, t(196) = 7.45, p < 0.001) as well as increased instances of social interaction (by phone, social media, or face-to-face, Fig. 2E, t(196) = 8.05, p < 0.001) compared to the start of the DS. Importantly, not only the number but the reported quality of social interactions (QSR) improved significantly (Fig. 2F, t(180) = 3.23, p < 0.001).

### Factors predicting well-being during the daily survey period

In order to identify the environmental parameters that impacted WB during the lockdown (DS period), the association between participants' environment, daily activities, and WB was examined. To this end, two hierarchical linear models (HLM) predicting DS WB were computed for the effects of the social environment and DS anxiety (model **A**; QSR, loneliness, num of people around, prosocial behavior, romantic relations, Table 4) and for daily events (model **B**; sleep, entertainment, physical activity, etc., Table 5). This analysis found that QSR was the strongest predictor of DS WB levels for the state QSR (daily effect), explaining nearly half the variance in DS WB (β = 0.45, p < 0.001), as well as trait QSR (overall period effect, β = 0.51, p < 0.001, Fig. 2G). Additionally, the number of people around and the number of social interactions had a small but significant positive state effect on DS WB levels (β = 0.07 and β = 0.08, respectively, p < 0.001). These variables did not show a trait effect. Furthermore, QSR mitigated the effect of DS anxiety on DS WB levels during the first lockdown. Across all DS anxiety levels, participants with high QSR had significantly higher DS WB than participants with low QSR (Fig. 2H, two-way ANOVA, F(192,1) = 29.92, p < 0.0001). Prosocial behavior had a small but significant state effect on DS WB. Interestingly, this effect stemmed from "giving" (β = 0.08, p < 0.001) but not "receiving" (β = 0.02, 0.12 p = 0.263) prosocial acts. Romantic relations had a strong trait effect (β = 0.11, p = 0.04) as well as state effect (β = 0.07, p < 0.001) on DS WB.

While social support was beneficial for WB, DS anxiety negatively predicted WB (Fig. 2I), with a significant state (β = − 0.18, p < 0.001) and trait (β = − 0.23, p < 0.001) effect on DS WB. Loneliness also had a significant state (β = − 0.18, p < 0.001) and trait (β = − 0.16, p < 0.001) effect on DS WB.

To determine whether any of the demographic variables could explain these effects, a linear regression was performed for each of these factors (QSR, DS anxiety, loneliness, prosocial behavior, romantic relations) with demographic variables entered as covariates (age, sex, marital status, economic status). This analysis found that the effect of all these factors on DS WB remained significant (p < 0.001), indicating that demographic information could not explain the reported effect (Table S2, supplementary materials).

An examination of non-social behaviors revealed a significant but small effect on DS WB (Table 5, model B). Engagement in sports (state β = 0.11, p = 0.05; trait β = 0.17, p < 0.001) or meditation (state β = 0.03 p = 0.05; trait β = 0.18, p = 0.03) significantly predicted increased DS WB. Dancing and/or listening to music also had a positive state effect on DS WB (β = 0.11, p < 0.001). The consumption of hedonic substances, including chocolate, alcohol, recreational drugs, and medication, had a significant state effect (β = 0.11, p < 0.001) on DS WB, but no trait effect was observed.

Unexpectedly, despite the strict stay-at-home mandate, a marked "weekend effect" for WB was observed on weekend days compared to weekdays. During the weekend, DS WB was significantly higher (Fig. 2J, ANOVA, F(1,4728) = 20.4, p < 0.001), and DS anxiety was significantly lower (Fig. 2K, ANOVA, F(1,4748) = 8.4, p = 0.004) than during weekdays. Additionally, substance use and sleep duration were significantly higher on the weekend (ANOVA, F(1,4771) = 32.7, p < 0.001; F(1,4755) = 23.8, p < 0.001, respectively). In parallel, a reduction in social interaction quantity (ANOVA, F(1,4728) = 20.4, p < 0.001) but an increase in quality (QSR) was observed (Fig. 2L, ANOVA, (F(1,4296) = 5.8, p = 0.02).

### Assessment of well-being a year later

**Figure 3:**
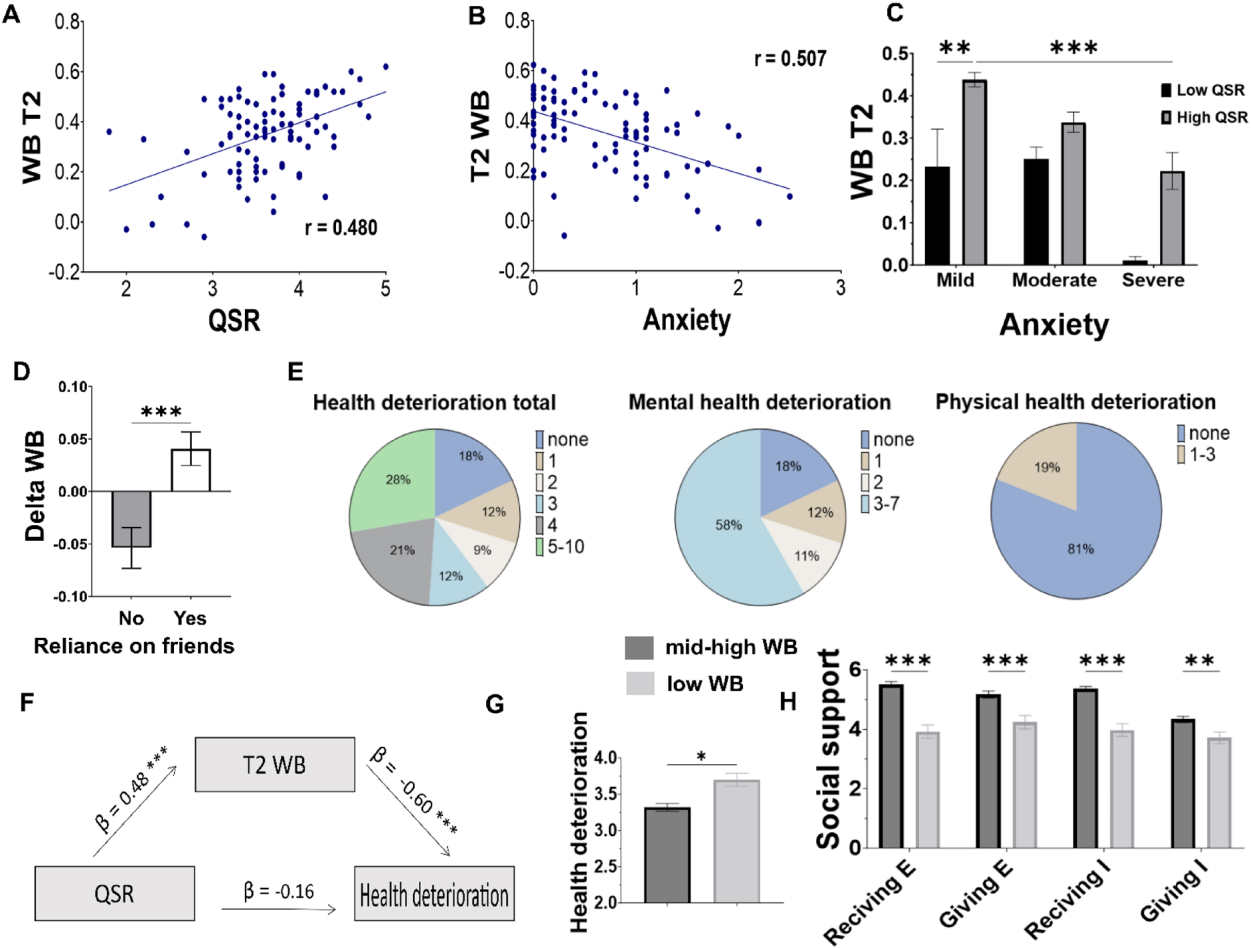
Factors associated with T2 WB and the health deterioration. (**A**) QSR was positively associated with T2 WB. (**B**) DS Anxiety was negatively associated with T2 WB. (**C**) High QSR participants reported higher T2 WB across all DS anxiety groups. (**D**) Significant difference in delta WB (T2-T1) was observed between participants who reported relying on friends vs. those who did not do so. (**E**) Pie charts present a breakdown of reported health deterioration by item for the total, mental and physical health items. (**F**) Pathway (mediation) analysis of the indirect effect of QSR on health. (**G**-**H**) The bottom third T1 WB participants presented significantly higher health deterioration and lower social support, giving, and receiving compared to the other participants. All error bars represent the standard error of the mean. *p < 0.05, **p < 0.001, ***p < 0.0001.

In May 2021, a year after the first survey, a subset of the participants (N = 94) responded to a follow-up questionnaire, termed "T2", which included the same T1 surveys and several additional questions (health and SSS, see “Materials and methods”). Similarly to T1, a composite WB score was constructed for T2 ("T2 WB", see “Materials and methods”). Analysis of T2 WB found no significant difference between T1 WB and T2 WB scores (p > 0.05). QSR significantly predicted higher T2 WB levels (Fig. 3A, β = 0.27, p < 0.001, model C, Table 6). In contrast, DS anxiety (Fig. 3B, β = − 0.36, p < 0.001) and loneliness (β = − 0.22, p = 0.05) predicted lower T2 WB. No other factors from the DS showed a significant effect (p > 0.05). Furthermore, as observed for DS WB, participants with high QSR levels reported higher T2 WB across DS anxiety levels (Fig. 3C, two-way ANOVA F (1, 85) = 17.52, p < 0.0001). To examine individual changes to WB, the difference between T1 and T2 WB was calculated ("delta WB"). A significant difference was observed between participants who reported reliance on friends in the past year to those who had not relied on friends (ANOVA, F(1,92) = 14.88, p < 0.001). While reliance on friends was associated with an improvement in WB, failing to do so was associated with a decrease in WB (Fig. 3D).

### Health deterioration a year later

Deterioration in mental and physical health (the "health deterioration score", see “Materials and methods”) was examined at T2. Most participants (82%) reported health deterioration in one or more of the 10 health items in T2, and about half (53%) reported a deterioration in three or more mental health items (Fig. 3E, Table 3). Notably, although our population was relatively young (mean age 31.5 ± 9.5 STD), 19% reported a deterioration in physical health, including frequent viral infections (10%), deterioration in pre-existing chronic diseases (8%), and abnormal blood-test values (8%).

A significant negative correlation was found between the health deterioration score and the T2 WB score (Pearson r = − 0.501, p < 0.001), indicative of a strong relationship between health and WB. To identify the predictors of health deterioration, the relationship between the DS factors and the health deterioration score was examined with simple linear models. This analysis revealed that DS anxiety was the strongest predictor of health deterioration (β = 0.41, t(92) = − 4.27 p < 0.001). Loneliness also had a significant effect (β = 0.26, t(92) = 2.60 p = 0.01), and QSR showed a marginal trend (β = − 0.17, t(91) = 1.17 p = 0.08). Next, a mediation analysis was conducted to examine the hypothesis that QSR indirectly influenced health. Indeed, QSR had an indirect negative effect on health deterioration a year later via its effect on T2 WB (ACME − 0.16 | [− 0.25, − 0.08] | p < 0.001). Accordingly, a positive association of QSR with T2 WB (β = 0.43 t(93) = 4.56 p < 0.001) and negative association of T2 WB with health deterioration (β = − 0.60, t(91) = − 6.18 p < 0.001) were observed. Thus, QSR levels reported during the DS period were indirectly beneficial for health at T2 (Fig. 3F).

Finally, the bottom third of participants scoring lowest on T1 WB ("low WB" group, n = 34/94) reported significantly more health deterioration compared to the other participants ("mid-high WB" group, ANOVA, F(1,92) = 15.1, p < 0.001, Fig. 3G). Importantly, the low WB group scored significantly lower in the SSS questionnaire at T2 for both receiving (F(1,92) = 55.1, p < 0.001) and giving (F(1,92) = 16.3, p < 0.001) emotional support, as well as receiving (F(1,92) = 52.8, p < 0.001) and giving (F(1,92) = 9.2, p = 0.003) instrumental support (Fig. 3H).

## Discussion

In this study, data was collected over a 6-week period, starting at the peak of the first COVID-19 lockdown in Israel, as well as a follow-up with the same participants a year later. We examined the factors affecting WB and health during this uniquely distressing period. Consistent with the literature worldwide^67,68^, as well as in Israel^3,8^, participants' WB was negatively impacted by anxiety during the pandemic. The most important factor that had a positive, enduring effect on WB and health was the quality of social interactions. QSR was not only the main predictor of WB levels during the lockdown, but it also had a protective role for health a year later. While other studies have previously reported a strong correlation between health deterioration and anxiety during the COVID-19 period^12,13,69,70^, here we demonstrate that this effect persists over the long term.

### State and trait effects reveal the enduring impact of quality of social relations on well-being

As many studies are cross-sectional, the repeated daily measurement of factors affecting WB using the ESM^40^ is a major advantage of this study, providing insight into the immediate and long-term effects of daily behaviors. The rich dataset collected daily during the peak of the first lockdown was analyzed using a mixed model framework^61^, allowing for two levels of analysis: the trait level effect (the person-average between-subject level) and the state level effect (the within-subject daily deviations from their overall mean) of multiple variables on WB levels. This strategy allowed us to identify which factors were beneficial only momentarily and which factors had a lasting impact on the DS period. This was expressed as a significant state effect but no enduring trait effect in certain variables. For example, the number of people around and social interactions had a minor state effect on WB but no trait effect, suggesting that social interactions in and of themselves are not necessarily beneficial. Indeed, it is important to keep in mind that not all social interactions are positive, and some could even be harmful. The stay-at-home policy enforced during the pandemic also increased domestic violence and problems within the family^68,71,72^. It is, therefore, not surprising that the number of social interactions was found to have less impact on WB than QSR, which indexes positive interactions. QSR was highly significant not only on the state level but also on the trait level, demonstrating the type of enduring effect that can have a significant impact on WB. It was also one of the few variables that predicted WB a year later and mitigated the effects of anxiety. As a highly social species, humans are dependent on social support not only for survival but also for emotional WB. The mitigating effect of social support on anxiety was established two decades ago^73,74^, and recent studies conducted during the pandemic period have yielded similar findings, showing that the quality rather than the quantity of social relationships had a significant impact on WB and mental health^30,32,35^. These findings converge with the data presented here to provide support for the importance of a positive social environment in maintaining health and WB, especially in distressing times like a global pandemic.

### Prosocial behavior, giving and receiving social support during the pandemic

Prosociality, specifically helping others, also positively affected WB. Interestingly, while performing a prosocial act had a small yet significant effect, being on the *receiving end* of one did not affect daily WB levels. While the effect of prosocial behavior was not an enduring one, expressed only as a state effect, it provides important evidence for the rewarding aspect of acting prosocially and indicates it has the potential to influence WB. When this point was explored further with the SSS questionnaire at T2, we found that participants with the lowest WB scores at T1 also reported significantly lower on giving and receiving prosocial acts at T2, and that reliance on friends for support during the lockdown was associated with individual improvement in WB a year later, demonstrating a long-term link between WB and a prosocial environment.

In line with these results, solidarity and helping behavior have been documented worldwide as the pandemic spread, including individual support for neighbors, establishing new organizations, and other prosocial activities^75–77^. Moreover, giving and receiving support was found to be linked to improved WB and mental health during the pandemic^29,30,30,78^. A cross-sectional study in the US and Canada found that reported formal volunteering and providing support was associated with higher positive affect^38^. Another multi-country survey of over 10,000 participants showed that self-reported prosocial behavior (e.g., helping friends or volunteering) was associated with better WB across regions^37^.

### The effects of social support on health

A vast body of research highlights the link between social support and improved health outcomes, including decreased inflammation, malignancy, and mortality^79,80^. Engaging in prosocial behavior has also been linked to enhanced WB and health^81^. One of the mechanisms underlying these protective health effects is that a positive social environment promotes a physiological 'safe' state and regulation of the stress response. According to the Social Safety Theory^82^, humans, as social beings, have evolved in such a way that the stress response is sensitive to social safety and belongingness. The brain is attuned to social cues that can modulate the hypothalamic–pituitary–adrenal (HPA) axis and the sympathetic nervous system to downregulate the stress response. A safe social network can buffer these reactions by recognizing 'safe' cues, which then moderate signals within these stress arousal systems, leading to a reduced peripheral response^83^. Empirical evidence supports these theories; an essential player in mediating HPA response is oxytocin, which is secreted in response to various affiliative social stimuli and can attenuate HPA axis responses^84,85^. This mechanism is shared to a certain extent across social species^86^. As stress is tightly linked to impaired immune function^87,88^, this provides a mechanism by which social support entails positive health effects. In summary, improved health outcomes observed here are posited to result from social facilitation of the 'safe' mode, which mitigates the negative effect of stress and anxiety.

### The role of daily activities on well-being during the pandemic

Not only did social factors affect WB, but other factors, such as engaging in sports, were also found to positively affect WB both on the state effect and the trait effect during the first lockdown. Participants who reported engaging in sports more frequently during the DS period had higher DS WB levels. This enduring effect, albeit not as significant as QSR, points to the potential of daily habits to positively impact WB. Similar findings were reported in other studies that showed the importance of physical activities and being outdoors for WB and mental health during the pandemic^17,46,89,90^.

Practicing meditation also had a small yet significant capacity to improve DS WB levels. Interestingly, the trait effect was more significant compared to a minor state effect, which could be explained by the beneficial additive effect of meditation. That is, the more often meditation was practiced, the stronger the impact on WB. A link between meditation and better health has been shown pre-pandemic as well as during the pandemic period^91–93^.

An increase in alcohol consumption and drug use has also been documented during the pandemic^94–96^. We found that consuming different hedonic substances—such as chocolate, drugs, alcohol, and medication—had a small positive association with DS WB levels. However, these behaviors had a state effect but not a trait effect on WB, suggesting the small improvement in WB on the day of consumption had no substantial enduring effect on WB, even tending to negatively impact overall DS WB, and may not be an effective strategy.

Another surprising effect we observed while visualizing the DS data is a "weekend effect". Despite the strict stay-at-home mandate, WB levels were higher on weekends than on weekdays, as was QSR, while anxiety was significantly lower. This is in line with studies conducted prior to the pandemic showing significantly more happiness, less anxiety, and better mood on weekends^97,98^. Notably, while QSR was significantly higher on the weekend, the number of social interactions was significantly lower. This provided further evidence for the primacy of the quality rather than quantity of social interactions as the main predictor of WB.

### Limitations of the study

The current study has several limitations. First, the study mainly consisted of young Israeli females. In recent studies, young females were found to be a vulnerable group and at risk for psychological distress during the COVID-19 period. Furthermore, Israeli subjects may have had a unique experience compared to other countries, for instance, the early adoption of a vaccine. This study is, therefore, especially relevant to this specific population and might be less generalizable for the general population. Yet, the many points of similarity with other studies reported above are encouraging and indicate that the behavior of Israeli participants is in line with global trends. Thus, the findings here are likely to be widely relevant and informative for policymakers, pending further research.

Another factor limiting the robustness of the results is a limited sample size. Out of the initial 206 participants, about half remained for the T2 time point a year later. Thus, T2 findings were based on 94 participants. However, a demographics analysis found no significant difference between the T1 and T2 distributions. Attrition between the two time points is a common occurrence in longitudinal studies. Yet, the longitudinal within-participant design is advantageous over a cross-sectional approach as it provides a unique perspective on the detailed experience of the participants and the impact of daily activity.

From a statistical point of view, in the analysis of DS factors predicting T2 WB, we focused on the means of the DS factors. In future studies, it would be beneficial to examine in-depth whether individual momentary variability, alongside 'trait' variables, may provide an additional perspective on long-term outcomes. This approach could provide insight into both stable traits and fluctuating states. Furthermore, the Cronbach's alpha scores for the T1 WB and T2 WB composites are 0.74 and 0.71, respectively. While these are in the lower acceptable range, they are nonetheless valid, especially considering the complexity of the WB concept. Another point to consider is that the models predicting the DS WB score did not account for the weekend effect, which might have strengthened the effects if it had been controlled for. Nonetheless, our findings did reveal a similar pattern during the weekend, characterized by an increase in WB and reduced DS anxiety levels. Concurrently, QSR was observed to be higher over the weekend. This pattern could imply that the improvement in WB is predominantly influenced by positive relationships over weekdays and weekends alike. Finally, it would have been ideal to be able to compare within-subject parameters to a pre-pandemic baseline level. As is, it is impossible to determine whether subjects who reported low WB had been vulnerable to begin with. However, while a baseline is structurally unobtainable in this study prompted by the pandemic onset, the within-subject ESM and longitudinal design provide increased robustness compared to a simple cross-sectional design.

### Conclusion

The current study found that anxiety and loneliness experienced during the first COVID-19 lockdown had a significant impact on WB and health that persisted even a year later. Positive social relationships and support played an important role in improving WB and protecting health. Specifically, a high relationship quality mitigated the negative effect of anxiety in the immediate and long term. While anxiety levels are hard to control, social support can be supplied by a community to those who lack it, thereby potentially thwarting long-term detriments to physical health. Hence, it is crucial to identify at-risk individuals as a preventative health screening, such as done with other physical risk factors (e.g., smoking). Focusing on others and improving relationships may be effective strategies for coping with global stressors such as pandemics and other crises.

### Supplementary Information

Supplementary Information.

## Data Availability

The data set generated for this study is available upon request from the corresponding author.
