# Supplementary material for: The effect of a prosocial environment on health and well-being during the first COVID-19 lockdown and a year later
Source: Sci Rep. 2024 Mar 19;14:6565. doi: 10.1038/s41598-024-56979-2 (PMC10951249; doi:10.1038/s41598-024-56979-2)
Supplement: Supplementary file 1 — Supplementary Information. [file 41598_2024_56979_MOESM1_ESM.docx]

| **Table S1.** Correlation between the DS's main factors and the total MHC, DASS, and MSPSS scores. | | | | | | | | |
| --- | --- | --- | --- | --- | --- | --- | --- | --- |
|  |  | **DS WB** | **Anxiety** | **Loneliness** | **QSR** | **MHC total** | **MSPSS total** | **DASS total** |
| **DS WB** | Pearson | 1.00 | -.419^**^ | -.564^**^ | .431^**^ | **.592^**^** | .427^**^ | -.341^**^ |
|  | Sig. (2-tailed) |  | 0.00 | 0.00 | 0.00 | **0.00** | 0.00 | 0.00 |
|  | N | 206 | 206 | 206 | 206 | 206 | 206 | 206 |
| **Anxiety** | Pearson | -.419^**^ | 1.00 | .358^**^ | -.168^*^ | -.360^**^ | -.166^*^ | **.432^**^** |
|  | Sig. (2-tailed) | 0.00 |  | 0.00 | 0.02 | 0.00 | 0.02 | **0.00** |
|  | N | 206 | 206 | 206 | 206 | 206 | 206 | 206 |
| **Loneliness** | Pearson | -.564^**^ | .358^**^ | 1.00 | -.355^**^ | -.475^**^ | **-.554^**^** | .396^**^ |
|  | Sig. (2-tailed) | 0.00 | 0.00 |  | 0.00 | 0.00 | **0.00** | 0.00 |
|  | N | 206 | 206 | 206 | 206 | 206 | 206 | 206 |
| **QSR** | Pearson | .431^**^ | -.168^*^ | -.355^**^ | 1.00 | .260^**^ | **.343^**^** | -.273^**^ |
|  | Sig. (2-tailed) | 0.00 | 0.02 | 0.00 |  | 0.00 | **0.00** | 0.00 |
|  | N | 206 | 206 | 206 | 206 | 206 | 206 | 206 |
| **MHC total** | Pearson | .592^**^ | -.360^**^ | -.475^**^ | .260^**^ | 1.00 | .549^**^ | -.537^**^ |
|  | Sig. (2-tailed) | 0.00 | 0.00 | 0.00 | 0.00 |  | 0.00 | 0.00 |
|  | N | 206 | 206 | 206 | 206 | 206 | 206 | 206 |
| **MSPSS total** | Pearson | .427^**^ | -.166^*^ | -.554^**^ | .343^**^ | .549^**^ | 1.00 | -.389^**^ |
|  | Sig. (2-tailed) | 0.00 | 0.02 | 0.00 | 0.00 | 0.00 |  | 0.00 |
|  | N | 206 | 206 | 206 | 206 | 206 | 206 | 206 |
| **DASS total** | Pearson | -.341^**^ | .432^**^ | .396^**^ | -.273^**^ | -.537^**^ | -.389^**^ | 1.00 |
|  | Sig. (2-tailed) | 0.00 | 0.00 | 0.00 | 0.00 | 0.00 | 0.00 |  |
|  | N | 206 | 206 | 206 | 206 | 206 | 206 | 206 |
| **. Correlation is significant at the 0.01 level (2-tailed). | | | | | | | | |
| *. Correlation is significant at the 0.05 level (2-tailed). | | | | | | | | |

| **Table S2**. Linear regression models predicting DS WB with demographic variables as covariates. | | | | | | |
| --- | --- | --- | --- | --- | --- | --- |
|  |  | **Unstandardized Coefficients** | | **Standardized Coefficients** | **t** | **Sig.** |
|  |  | **B** | **Std. Error** | **Beta** |  |  |
| Model 1 | (Constant) | 4.29 | 0.56 |  | 7.70 | **0.00** |
|  | Sex | 0.26 | 0.19 | 0.09 | 1.35 | 0.18 |
|  | Age | 0.00 | 0.01 | 0.02 | 0.32 | 0.75 |
|  | Marital.st | 0.15 | 0.09 | 0.13 | 1.71 | 0.09 |
|  | Economic.st | 0.19 | 0.10 | 0.12 | 1.89 | 0.06 |
|  | **Anxiety** | -0.63 | 0.10 | -0.40 | -6.16 | **0.00** |
| R2 Conditional = 0.220, R2 marginal = 0.200 | | | | | | |
| Model 2 | (Constant) | 5.09 | 0.53 |  | 9.55 | **0.00** |
|  | Sex | 0.04 | 0.18 | 0.01 | 0.21 | 0.84 |
|  | Age | 0.00 | 0.01 | -0.01 | -0.09 | 0.93 |
|  | Marital.st | 0.03 | 0.08 | 0.02 | 0.34 | 0.74 |
|  | Economic.st | 0.16 | 0.09 | 0.10 | 1.70 | 0.09 |
|  | **Loneliness** | -2.49 | 0.28 | -0.54 | -8.75 | **0.00** |
| R2 Conditional = 0.329, R2 marginal = 0.313 | | | | | | |
| Model 3 | (Constant) | 2.47 | 0.57 |  | 4.35 | **0.00** |
|  | Sex | 0.03 | 0.19 | 0.01 | 0.16 | 0.87 |
|  | Age | 0.00 | 0.01 | -0.01 | -0.12 | 0.90 |
|  | Marital.st | 0.10 | 0.09 | 0.09 | 1.19 | 0.24 |
|  | Economic.st | 0.32 | 0.10 | 0.21 | 3.25 | **0.00** |
|  | **QSR** | 0.47 | 0.07 | 0.42 | 6.66 | **0.00** |
| R2 Conditional = 0.240, R2 marginal = 0.221 | | | | | | |
| Model 4 | (Constant) | 3.39 | 0.58 |  | 5.79 | **0.00** |
|  | Sex | 0.10 | 0.20 | 0.03 | 0.48 | 0.63 |
|  | Age | 0.00 | 0.01 | -0.01 | -0.16 | 0.87 |
|  | Marital.st | 0.10 | 0.10 | 0.08 | 1.05 | 0.30 |
|  | Economic.st | 0.35 | 0.11 | 0.23 | 3.32 | **0.00** |
|  | **Prosocial giving** | 0.95 | 0.27 | 0.25 | 3.54 | **0.00** |
| R2 Conditional = 0.125, R2 marginal = 0.103 | | | | | | |
| Model 4 | (Constant) | 3.46 | 0.58 |  | 6.00 | **0.00** |
|  | Sex | 0.13 | 0.20 | 0.05 | 0.67 | 0.50 |
|  | Age | 0.00 | 0.01 | 0.02 | 0.20 | 0.84 |
|  | Marital.st | 0.14 | 0.09 | 0.11 | 1.45 | 0.15 |
|  | Economic.st | 0.33 | 0.10 | 0.22 | 3.20 | **0.00** |
|  | **romantic relations** | 1.98 | 0.49 | 0.27 | 4.07 | **0.00** |
| R2 Conditional = 0.142, R2 marginal = 0.120 | | | | | | |

**R script:**

library(ggcorrplot)

library(dplyr)

library(tidyr)

library(broom)

library(psych)

library(parameters)

library(tidyverse)

library(lmerTest)

library(parameters)

library(performance)

library(emmeans)

library(ggeffects)

library(ggeffects)

library(correlation)

library(finalfit)

library(mice)

library(ggforce)

library(ggplot2)

library(lme4)

library(lmerTest)

library(datawizard)

#home

setwd("C:/Users/esthe/Dropbox/TAU/Humans/Happyapp/Analysis/R - Files/Matan")

#work

setwd("C:/Users/User/Dropbox/TAU/Humans/Happyapp/Analysis/R - Files/Matan")

# APP ---------------------------------------------------------------------

#app's raw data +fs

raw_app_data <- read.csv("Data/app_15.12.csv")

#sport = C6

fs_app_data <- raw_app_data |>

mutate(

across(Q1:Q13, .fns = as.numeric),

Date = as.Date(timestamp2, format = "%d/%m/%Y"),

Wellbeing = Q1 + Q5 + Q6,

Q8 = 1-Q8,

Q9 = 1-Q9,

sport = C6, # addetive sport effect

substance = C7 + C15 + C11 + C12 ) |>

filter( Date != as.Date("13/04/2020", format = "%d/%m/%Y"),

Date != as.Date("28/05/2020", format = "%d/%m/%Y") ) |>

group_by(subjectid) |>

filter(n() > 5) |>

ungroup() |>

mutate(subjectid = factor(subjectid)) |>

mutate(

Q13 = ifelse(Date == "2020-04-14" | Date == "2020-04-15" | Date == "2020-04-16", NA, Q13))

# fs with the dif between within, yesterday and between

fs_q89 <- fs_app_data |>

group_by(subjectid) |>

mutate(

across(c(starts_with("Q"), starts_with("C"), substance, Wellbeing, sport), .fns = lag, n = 1, .names = "{.col}_lasttime")

) |>

ungroup() |>

drop_na(Wellbeing, starts_with("Q"), starts_with("C")) |>

mutate(

# Today

datawizard::demean(cur_data(), group = "subjectid",

select = c("Q1", "Q2", "Q3", "Q4", "Q5", "Q6", "Q8", "Q9", "Q10", "Q12", "Q13", "Wellbeing")),

datawizard::demean(cur_data(), group = "subjectid",

select = c("C1", "C2", "C3", "C4", "C5","C6", "C7", "C8", "C9", "C10", "C11", "C12", "C13", "C14", "C15", "C17", "sport", "substance")),

# Yesterday

datawizard::demean(cur_data(), group = "subjectid",

select = paste0(c("Q1", "Q2", "Q3", "Q4", "Q5", "Q6", "Q8", "Q9", "Q10", "Q12", "Q13" ,"Wellbeing"), "_lasttime")),

datawizard::demean(cur_data(), group = "subjectid",

select = paste0(c("C1", "C2", "C3", "C4", "C5", "C6", "C7", "C8", "C9", "C10", "C11", "C12", "C13", "C14", "C15", "C17", "sport", "substance"), "_lasttime")),

# Factors

across(c(Q4, Q8, Q9), factor, labels = c("No", "Yes")),

across(c(Q4_lasttime, Q8_lasttime, Q9_lasttime), factor, labels = c("No", "Yes")) )

#new varible delatWB

Q_data_wellbeing$deltaWB <-Q_data_wellbeing$WBT5-Q_data_wellbeing$WBT1

# Multiple reg - Qs' & WB app ------------------------------------------------

model_a <- lmer(Wellbeing ~ Q4_between + Q2_between + Q8_between + Q9_between + Q10_between + Q3_between + Q13_between + C14_between

+ C14_within + Q4_within + Q2_within + Q8_within + Q9_within + Q10_within + Q3_within + Q13_within +

(Q4_within + Q2_within + Q8_within + Q9_within + Q10_within + Q3_within + Q13_within | subjectid),

data = fs_q89)

summary(model_a) # no 0 variance componants (warning is false)

performance::check_collinearity(model_a)

model_parameters(model_a, standardize = "pseudo", ci_method = "s")

c(conditional = cor(fs_q89$Wellbeing, predict(model_a))^2,

marginal = cor(fs_q89$Wellbeing, predict(model_a, re.form = NA))^2)

r2(model_a)

summary(model_a)

multiple5_sum <- summary (model_a)

multiple5_tab <- model_parameters(model_a, standardize = "pseudo", ci_method = "s")

write.csv(multiple5_tab,"Data Processed/Models/m_multi_par_table.csv")

ggpredict(model_a,"Q13_within",) |> plot() +

coord_cartesian(ylim = c(0, 4))

ggpredict(model_a,"Q3_within",) |> plot() +

coord_cartesian(ylim = c(0, 4))

ggpredict(model_a,"Q10_within",) |> plot() +

coord_cartesian(ylim = c(0, 4))

ggpredict(model_a,"Q4_within",) |> plot() +

coord_cartesian(ylim = c(0, 4))

ggpredict(model_a,"Q2_within",) |> plot() +

coord_cartesian(ylim = c(0, 4))

ggpredict(model_a,"Q8_within",) |> plot() +

coord_cartesian(ylim = c(0, 4))

ggpredict(model_a,"Q9_within",) |> plot() +

coord_cartesian(ylim = c(0, 4))

capture.output(summary(model_a), file = "Data Processed/Models/manuscript/sumodel_a.txt")

capture.output(model_parameters(model_a, standardize = "pseudo", ci_method = "s"), file = "Data Processed/Models/manuscript/par_multiple5.txt")

# Multiple reg C and WB -----------------------------------------------------------

#all sports C's___________________________________________

#substance use + sports + otherC's

model_b <- lmer(Wellbeing ~sport_between + substance_between + Q12_between +

C8_between + C9_between + C10_between + C13_between +

sport_within + substance_within + Q12_within+

C8_within + C9_within + C10_within + C13_within +

(sport_within + substance_within + C8_within + C9_within + C10_within + C13_within + Q12_within|subjectid ),

data = fs_q89)

model_parameters(model_b, standardize = "pseudo", ci_method = "s")

model_performance(model_b)

performance::check_collinearity(model_b)

r2(model_b)

capture.output(summary(model_b), file = "Data Processed/Models/manuscript/model_b.txt")

capture.output (model_parameters(model_b , standardize = "pseudo", ci_method = "s"),

file = "Data Processed/Models/manuscript/par_multiCC_2.txt")

multiple6_tab <- model_parameters(model_b, standardize = "pseudo", ci_method = "s")

write.csv(multiple6_tab,"Data Processed/Models/m_multi_par_table3.csv")

c(conditional = cor(fs_q89$Wellbeing, predict(model_b))^2,

marginal = cor(fs_q89$Wellbeing, predict(model_b, re.form = NA))^2)

ggpredict(model_b,"substance_within",) |> plot() +

coord_cartesian(ylim = c(0, 4))

ggpredict(model_b,"substance_between",) |> plot() +

coord_cartesian(ylim = c(0, 4))

ggpredict(model_b,"sport_within",) |> plot() +

coord_cartesian(ylim = c(0, 4))

ggpredict(model_b,"C14_within",) |> plot() +

coord_cartesian(ylim = c(0, 4))

# Corr + Pivot longer for app daily graphs ---------------------------------------------------

#daily_graphs

corr_q <- correlation(fs_app_data, select =c("Wellbeing","Q2","Q3","Q4","Q8","Q9","Q10","Q12","Q13"),

p_adjust = "bonferroni")

summary(corr_q)

capture.output(summary(corr_q), file = "Data Processed/Models/manuscript/corr_q.txt")

ggcorrplot(corr_q, method ="square")

ggcorrplot(Qs, method = "circle" )

fs_app_data_long |>

mutate(scale_type = name %in% c()) |>

ggplot(aes(x = Date, y = value, color = name)) +

facet_grid(rows = vars(scale_type), scales = "free_y") +

stat_summary() +

stat_summary(geom = "line") +

scale_fill_distiller(type = "div", palette = 1,

limits = c(0, 1),

oob = scales::squish) +

scale_x_date(date_breaks = "1 day") +

theme_bw() + theme(axis.text.x = element_text(angle = 90))

fs_app_data_longC_2<- fs_app_data |>

select(subjectid, Date, Wellbeing, sport, substance,C8, C9, C10, C13, C14) |>

change_scale(Wellbeing, to = c(0, 1), range = c(0, 3))|>

pivot_longer(cols = c(Wellbeing, sport, substance, C8, C9, C10, C13, C14))

fs_app_data_longC_2 |>

mutate(scale_type = name %in% c()) |>

ggplot(aes(x = Date, y = value, color = name)) +

facet_grid(rows = vars(scale_type), scales = "free_y") +

stat_summary() +

stat_summary(geom = "line") +

scale_fill_distiller(type = "div", palette = 1,

limits = c(0, 1),

oob = scales::squish) +

scale_x_date(date_breaks = "1 day") +

theme_bw() + theme(axis.text.x = element_text(angle = 90))

# Delta and T5 ------------------------------------------------------------

data_demo <- readRDS("Data Processed/data_demo.rds")

MHC_data <- readRDS("Data Processed/MHC_data.rds") |> filter(Questionnaire.no %in% c(1, 5))

DASS_data <- readRDS("Data Processed/DASS_data.rds") |> filter(Questionnaire.no %in% c(1, 5))

MSPSS_data <- readRDS("Data Processed/MSPSS_data.rds") |> filter(Questionnaire.no %in% c(1, 5))

AppMeans <- readRDS("Data Processed/AppMeans.rds")

T5_data <- readRDS("Data Processed/T5_data.rds")

SS2_data <- readRDS("Data Processed/SS2_data.rds")

Q_data <- data_demo |>

select(-Questionnaire.no) |>

full_join(AppMeans, by = c(Subject.ID = "subjectid")) |>

full_join(MHC_data, by = "Subject.ID") |>

full_join(DASS_data, by = c("Subject.ID", "Questionnaire.no")) |>

full_join(MSPSS_data, by = c("Subject.ID", "Questionnaire.no")) |>

full_join(T5_data |> select(-Questionnaire.no), by = "Subject.ID") |>

full_join(SS2_data |> select(-Questionnaire.no), by = "Subject.ID")

Q_data |>

select(Questionnaire.no, DASS_total, MHC_total, MSPSS_total) |>

mutate(DASS_total = -DASS_total) |>

group_by(Questionnaire.no) |>

summarise(alpha = psych::alpha(cur_data())$total$std.alpha)

Q_data_wellbeing <- Q_data |>

group_by(Questionnaire.no) |> mutate(

# DASS_total_r = -DASS_total,

# Wellbeing = across(c(DASS_total_r, MHC_total, MSPSS_total), scale) |>

# rowMeans(na.rm = TRUE)

DASS_total_rs = change_scale(-DASS_total, to = c(0, 1), range = c(0, 60)),

MHC_total_s = change_scale(MHC_total, to = c(0, 1), range = c(1, 6)),

MSPSS_total_s = change_scale(MSPSS_total, to = c(0, 1), range = c(1, 7)),

WellbeingT = across(c(DASS_total_rs, MHC_total_s, MSPSS_total_s)) |>

rowMeans(na.rm = TRUE)

) |>

mutate(Economic_damage = as.numeric(Economic_damage)) |>

ungroup() |>

select(-starts_with("DASS"), -starts_with("MSPSS"), -starts_with("MHC"), -starts_with("Timestamp")) |>

pivot_wider(values_from = WellbeingT, names_from = Questionnaire.no, names_prefix = "WBT") |>

drop_na(WBT5) |>

mutate(

across(c(Post_2, Health_post),

\(x) Hmisc::impute(x, fun = mean) |> as.numeric()) )

# T5WB models -------------------------------------------------------------

model_c <- lm(WBT5 ~ Q2 + Q3 + Q4 + Q8 + Q9 + +Q12 + Q10 + Q13

, data = Q_data_wellbeing )

check_collinearity(model_c)

model_parameters(model_c, standardize = "basic")

model_performance(model_c)

r2(model_c)

capture.output(model_parameters(model_c, standardize = "pseudo", ci_method = "s"), file = "Data Processed/Models/manuscript/model_c.txt")

# Delta WB -----------------------------------------------------------------

m1wb <- lm(deltaWB ~ Q2 + Q3 + Q4 + Q10 + Q13 + Q12

, data = Q_data_wellbeing )

model_parameters(m1wb, standardize = "basic")

check_collinearity(m1wb)

model_performance(m1wb)

ggpredict(m1wb,"Q2",) |> plot() +

coord_cartesian(ylim = c(0, 0.1))

m2wb <- lm(WBT5 ~ WBT1 + Q2 +Q13*Q2

, data = Q_data_wellbeing )

model_parameters(m2wb, standardize = "basic")

ggpredict(m2wb,"Q2",) |> plot() +

coord_cartesian(ylim = c(0, 0.1))

check_collinearity(m2wb)

model_performance(m2wb)

ggpredict(m2wb,"Q2",) |> plot() +

coord_cartesian(ylim = c(0, 0.6))

capture.output(summary(m2wb), file = "Data Processed/Models/manuscript/m2wb.txt")

m3wb <- lm(deltaWB ~ Res_friends + Res_family + Res_job +

Res_socialmedia + Res_entertainment,

data = Q_data_wellbeing)

model_parameters(m3wb, standardize = "basic")

model_performance(m3wb)

ggemmeans(m3wb, "Res_friends") |> plot()

check_collinearity(m3wb)

capture.output(summary(m3wb), file = "Data Processed/Models/manuscript/m3wb.txt")

# health post --------------------------------------------------------------

m1hp <- lm(Health_post ~ Q2 + Q3 + Q4 + Q10 + Q13 + Q12

, data = Q_data_wellbeing)

model_parameters(m1hp, standardize = "basic")

check_collinearity(m1hp)

model_performance(m1hp)

ggpredict(m1hp,"Q2",) |> plot() +

coord_cartesian(ylim = c(2, 5))

#capture.output(summary(m1hp), file = "Data Processed/Models/manuscript/m1hp.txt")

m2hp <- lm(Health_post ~ Q2

, data = Q_data_wellbeing)

model_parameters(m2hp, standardize = "basic")

model_performance(m2hp)

#capture.output(summary(m2hp), file = "Data Processed/Models/manuscript/m2hp.txt")

ggpredict(m2hp,"Q2",) |> plot() +

coord_cartesian(ylim = c(2, 5))

# Path analysis -----------------------------------------------------------

#first use this:

library(mediation)

# Q13

m_health2 <- lm(Health_post ~ WBT5 + Q13,

data = Q_data_wellbeing)

m_WB2 <- lm(WBT5 ~ Q13,

data = Q_data_wellbeing)

med2 <- mediate(m_WB2, m_health2,

treat = "Q13",

mediator = "WBT5",

outcome = "Health_post")

model_parameters(m_WB2, standardize = "basic")

model_parameters(m_health2, standardize = "basic")

model_performance(m_health2)

summary(m_health2)

model_parameters(m_WB2, standardize = "basic")

model_performance(m_WB2)

summary(m_WB2)

model_parameters(med2)

summary(med2)

capture.output(summary(med2), file = "Data Processed/Models/manuscript/sum_med2_Q13.txt")

#Q4-->WB-->health_post----------------------------

m_health4<- lm(Health_post ~ Q4 + WBT5,

data = Q_data_wellbeing)

m_WB4 <- lm(WBT5 ~ Q4,

data = Q_data_wellbeing)

model_performance(m_health4)

model_performance(m_WB4)

model_parameters(m_health4, standardize = "basic")

model_parameters(m_WB4, standardize = "basic")

med4 <- mediate(m_WB4, m_health4,

treat = "Q4",

mediator = "WBT5",

outcome = "Health_post")

model_parameters(med4)

capture.output(summary(med4), file = "Data Processed/Models/manuscript/sum_med4_Q4.txt")

m_health4a<- lm(Health_post ~ Q4,

data = Q_data_wellbeing)

model_performance(m_health4a)

model_parameters(m_health4a, standardize = "basic")

# Q2 + WBT5 - health ------------------------------------------------------

m_healthq2<- lm(Health_post ~ Q2 + WBT5,

data = Q_data_wellbeing)

model_performance(m_healthq2)

model_parameters(m_healthq2, standardize = "basic")

m_q2 <- lm(WBT5 ~ Q2,

data = Q_data_wellbeing)

model_parameters(m_q2, standardize = "basic")

model_performance(m_q2)

medq2 <- mediate(m_healthq2, m_q2,

treat = "Q2",

mediator = "WBT5",

outcome = "Health_post")

model_parameters(medq2)

capture.output(summary(medq2), file = "Data Processed/Models/manuscript/sum_medq2.txt")
